# Connecting optical remote sensing of plant photosynthesis with biogenic volatile organic compound emissions

**DOI:** 10.1111/nph.70504

**Published:** 2025-08-31

**Authors:** Chao Zhang, Jaana Bäck, Josep Peñuelas, Daijun Liu, Iolanda Filella, Albert Porcar‐Castell, Jon Atherton

**Affiliations:** ^1^ Optics of Photosynthesis Laboratory, Institute for Atmospheric and Earth System Research (INAR)/Forest Sciences, Viikki Plant Science Centre (ViPS) University of Helsinki 00014 Helsinki Finland; ^2^ Institute for Atmospheric and Earth System Research/Forest Sciences, Faculty of Agriculture and Forestry University of Helsinki 00014 Helsinki Finland; ^3^ Global Ecology Unit CREAF‐CSIC‐UAB Bellaterra Catalonia 08193 Spain; ^4^ CREAF Campus UAB, Cerdanyola del Vallès Catalonia 08193 Spain; ^5^ Department of Botany and Biodiversity Research University of Vienna Rennweg 14, 8 1030 Vienna Austria

**Keywords:** carotenoids, Chl/carotenoid index, isoprene, monoterpenes, photochemical reflectance index, photosynthesis, sun‐induced fluorescence, xanthophyll cycle

## Abstract

Plant biogenic volatile organic compounds (BVOCs) play a critical role in atmospheric chemistry by forming ozone and secondary organic aerosols, making them key agents in regulating air quality and influencing climate. However, current models usually rely on limited site‐specific data and indirect inputs, introducing significant uncertainties in BVOC predictions. We propose remote sensing of photosynthetic optical signals, such as the carotenoid‐sensitive photochemical reflectance index (PRI) and Chl/carotenoid index (CCI) and sun‐induced fluorescence (SIF), to help reduce these uncertainties. These indices are functionally linked, albeit indirectly for SIF, to isoprenoid BVOC emissions via carotenoid biosynthesis. In this Viewpoint, we explore the potential of this connection to estimate and constrain BVOC emissions at multiple scales. We synthesize key aspects, recent advances, and research uncertainties, and propose empirical and scaling roadmaps for integrating optical signals with BVOCs, highlighting their connectivity under abiotic stress (e.g. drought, heat) and across seasonal dynamics. This integration represents a critical step toward reducing model uncertainties, improving large‐scale BVOC monitoring, and enhancing our understanding of their role in atmospheric chemistry and climate. By providing a more comprehensive framework for linking plant physiological processes to atmospheric chemistry, this approach strengthens our ability to predict ecosystem responses to climate change.

## Introduction

Biogenic volatile organic compounds (BVOCs), primarily emitted by plants (Laothawornkitkul *et al*., 2009), play a key role in atmospheric processes, influencing air quality, cloud formation, and climate change (Kulmala *et al*., 2014; Scott *et al*., 2018). They are major sources of atmospheric aerosols and tropospheric ozone (Peñuelas & Staudt, 2010; Palmer *et al*., 2022) and contribute to radiative forcing through aerosol scattering and the formation of cloud droplets (Rap *et al*., 2018; Weber *et al*., 2022). By consuming hydroxyl radicals (OH) to form secondary organic aerosols and ozone, BVOCs decrease the capacity of tropospheric OH to undergo photochemical reactions with methane, extending methane's atmospheric lifetime and further accelerating global warming (Weber *et al*., 2022). Given their significant role in atmospheric processes, it is essential to understand how BVOC emissions respond to climate change.

As global temperatures rise due to elevated atmospheric CO_2_ concentrations, BVOC emissions are generally expected to increase (Szopa *et al*., 2021). However, the amount of growth is uncertain due to the increasing occurrence of extreme events like droughts and heatwaves (Yuan *et al*., 2023; Zhou *et al*., 2023), which can either enhance or reduce emissions (Wang *et al*., 2024; Bourtsoukidis *et al*., 2024b). These variations are influenced by plant physiological status and stress severity and duration. Climate‐mediated factors such as enhanced enzymatic activity, higher tissue vapor pressure, and reduced diffusion resistance drive BVOC emissions up (Loreto & Schnitzler, 2010; Niinemets *et al*., 2010; Peñuelas & Staudt, 2010). Conversely, elevated CO_2_ reduces emissions by limiting substrate availability (Loreto & Schnitzler, 2010; Sahu *et al*., 2023), while severe heat and drought stress can inhibit emissions via photoinhibition and enzymatic limitations (Possell & Loreto, 2013; Bourtsoukidis *et al*., 2024b). Therefore, accurately quantifying BVOC emissions under variable environmental conditions remains challenging.

Although BVOCs play critical roles in the atmosphere, methods for directly measuring their emissions at regional and global scales are lacking. Isoprene, the most abundant BVOC, accounting for 50–60% of global BVOC emissions, can be indirectly estimated via satellite retrievals of its oxidation product, formaldehyde (HCHO) (Rahman, 2023), but isoprene contributes only *c*. 30% of global HCHO production (Stavrakou *et al*., 2009). Of note, satellite measurements from the Cross‐track Infrared Sounder (CrIS) have recently improved global isoprene monitoring (Fu *et al*., 2019; Wells *et al*., 2020, 2022; Palmer *et al*., 2022). Global measurements of other key BVOCs, such as monoterpenes, remain unavailable, so BVOC emissions are typically characterized using models, such as MEGAN (Model of Emissions of Gases and Aerosols from Nature), which describes plant functional type‐specific emission sensitivities to temperature and light (Guenther, 2013). Despite advancements toward physiological reality, discrepancies have been highlighted between modeled and observed emissions (Guenther *et al*., 2012; Grote *et al*., 2013; Niinemets *et al*., 2013), due to reliance on indirect inputs, complex environmental stress effects, seasonal dynamics of plant photosynthetic activity, and limited observation data (Guenther *et al*., 2012; Grote *et al*., 2013; DiMaria *et al*., 2023; Wang *et al*., 2024).

The two most abundant plant‐emitted BVOCs, based on estimated annual global emissions, are isoprene and monoterpenes (Guenther *et al*., 2012). Both share the isoprene unit as their basic building block, and belong to the large family of isoprenoids (Peñuelas & Munné‐Bosch, 2005). Isoprene and monoterpenes serve photoprotective roles, such as quenching reactive oxygen species that can form under excess radiation, due to the conjugated double‐bond structure facilitating energy transfer and heat dissipation (Peñuelas & Munné‐Bosch, 2005). Carotenoids are another class of photoprotective isoprenoid compounds, primarily found in chloroplasts. As foliar pigments, carotenoids influence the optical properties of vegetation, which can be remotely sensed (Gamon *et al*., 2016).

This Viewpoint focuses on the connection between isoprenoid BVOC emissions and the remote sensing of carotenoids, as well as related physiological processes, such as carotenoid‐based vegetation indices and Chl fluorescence. In this context, we use the term BVOC primarily to refer to volatile isoprenoids linked to photosynthesis. However, there are many other BVOCs – such as green leaf volatiles – that play important roles in plant signaling and defense (Matsui & Engelberth, 2022). These often originate from distinct, nonphotosynthetic pathways and are often more challenging to detect via photosynthetic optical signals.

**Fig. 1:**
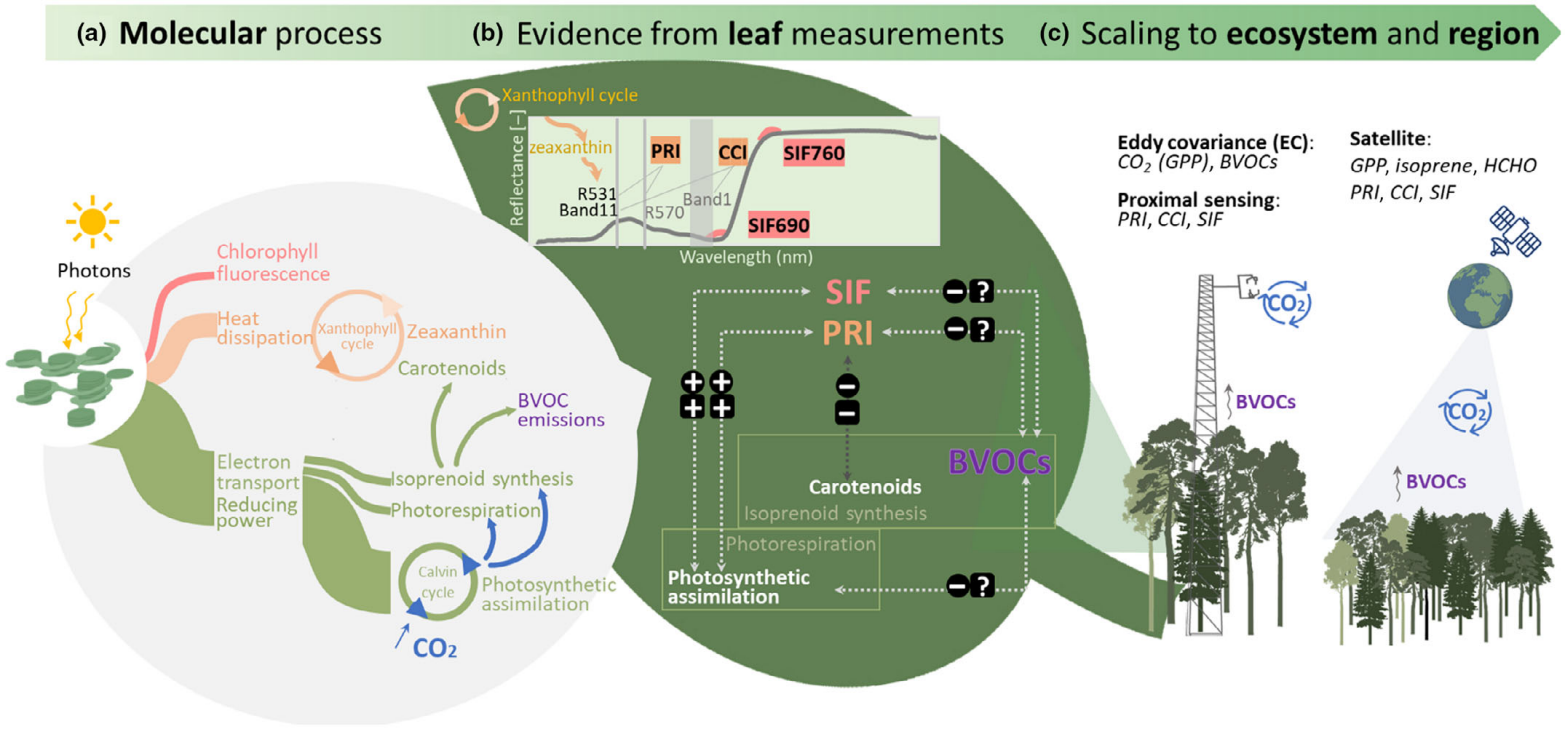
A schematic diagram illustrating the use of photosynthetic optical signals to measure plant biogenic volatile organic compound (BVOC) emissions at (a) molecular, (b) leaf, and (c) ecosystem and regional scales. The molecular process diagram in (a) is adapted from Filella *et al*. (2018). CCI, Chl/carotenoid index; PRI, photochemical reflectance index; SIF, sun‐induced fluorescence; SIF690 and SIF760, SIF emissions peaks at 690 and 760 nm, respectively; GPP, gross primary productivity; HCHO, formaldehyde. The molecular and leaf scale diagrams illustrate the functional linkages between BVOC emissions, PRI, Chl fluorescence, and photosynthetic assimilation. The leaf scale diagram also shows the potential correlations between BVOC emissions, PRI, CCI, SIF, and photosynthetic assimilation, with (+), (−), and (?) indicating positive, negative, and unknown correlations, respectively. Circle represents correlations under no or mild stress conditions, while rectangle indicates the correlations under severe stress conditions.

The photochemical reflectance index (PRI) and sun‐induced fluorescence (SIF) offer promising approaches to bridging large‐scale knowledge gaps in BVOC emissions, as both signals can potentially be detected remotely from space. PRI is closely linked to BVOC emissions through the *in vivo* formation of carotenoids, which share the same biosynthetic pathway as BVOCs, and through its relationship with photosynthetic light use efficiency (LUE) (Fig. 1a) (Peñuelas *et al*., 2015). Although widely applied at the leaf and canopy scales on shorter, daily timescales, only limited evidence suggested that seasonal changes in LUE could be detected from artificial satellite PRI (Zhang *et al*., 2016). Hence, a revised formulation, the Chl/carotenoid index (CCI) emerged as a pragmatic successor to PRI that better captures photosynthetic phenology from space (Gamon *et al*., 2016). Studies (Supporting Information Table S1) have shown that PRI or CCI are effective indicators of BVOC emissions on both short‐term (Peñuelas *et al*., 2013; Balzarolo *et al*., 2018) and long‐term (Harris *et al*., 2016; Filella *et al*., 2018) scales, especially in response to environmental stresses such as drought (Peñuelas *et al*., 2013; Filella *et al*., 2018). In addition to the carotenoid indices, remotely sensed SIF has been used to estimate HCHO and isoprene emissions (Table S1), via the correlation between SIF and gross primary productivity (GPP), that is ecosystem‐scale photosynthesis (Zheng *et al*., 2017; Zhao *et al*., 2024).

These studies have opened the door to the possibility that optical remote sensing of plant properties may be used to better constrain global emission estimates of BVOCs. But there are numerous uncertainties that need to be addressed before this can be achieved (see Current uncertainties and future empirical research directions), and a robust program of plant science research is essential to do so. Therefore, the aim of this Viewpoint is to advocate for an acceleration of empirical research linking BVOC emissions to the optical remote sensing of photosynthesis. First, we review the background of BVOC emissions from the leaf level to the global scale and present the functional connections between isoprenoid BVOCs and foliar optical properties. Next, we highlight key uncertainties and open research questions, primarily related to these functional links. Finally, we conclude the Viewpoint with a brief roadmap outlining how this research could contribute to better constraining global emission estimates.

## BVOC emission estimation methodologies: from leaves to the globe

At the leaf level, BVOCs are measured directly *in situ* using enclosure systems (Niinemets *et al*., 2011). Enclosure measurements can provide excellent temporal resolution (Niinemets *et al*., 2011; Li *et al*., 2019); however, upscaling is difficult due to the large variability in emissions between plant species (Niinemets *et al*., 2013; Bourtsoukidis *et al*., 2024a), landscape types (Guenther *et al*., 2012), and anthropogenic land cover changes (Unger, 2014). Ecosystem emission (and deposition) fluxes can be measured using the eddy covariance method, a micrometeorological technique (Sarkar *et al*., 2020) that is typically applied to relatively homogenous footprints.

Methods for measuring BVOC emissions at larger scales, such as regional and global scales, are lacking. Instead, remote sensing retrievals of the isoprene oxidation product formaldehyde (HCHO) from platforms such as the Global Ozone Monitoring Experiment (GOME) and the TROPOspheric Monitoring Instrument (TROPOMI) have stood in as a top‐down, indirect proxy for isoprene emissions at such scales (Rahman, 2023). However, sources such as vegetation fires can significantly increase HCHO levels (Stavrakou *et al*., 2009), particularly with the increasing frequency of extreme drought and heatwaves (Jones *et al*., 2022; Morfopoulos *et al*., 2022). Specifically, a study by Zhao *et al*. (2022) found that BVOCs contributed < 10% to HCHO production during wildfire activity in the Alaskan summer, with background HCHO and wildfire emissions accounting for over 70%. This highlights the limitations of using HCHO as a reliable proxy for isoprene emissions.

Recent satellite measurements from CrIS (launched in 2011) provide a significant advancement in monitoring isoprene emissions globally (Fu *et al*., 2019; Wells *et al*., 2020, 2022; Palmer *et al*., 2022). The retrieval of isoprene from CrIS is based on its absorption cross‐section in the infrared spectrum (Brauer *et al*., 2014) and has been successfully applied at both regional (Fu *et al*., 2019; Wells *et al*., 2022) and global (Wells *et al*., 2020) scales to examine seasonal‐to‐interannual changes in isoprene, with validation using *in situ* data and model outputs. The potential interferences of isoprene with nonisoprene species such as H_2_O and other VOCs have also been discussed (Fu *et al*., 2019), with monoterpene interference found to have a relatively small impact (Wells *et al*., 2022). This information is crucial for interpreting the validity of CrIS‐retrieved isoprene data. However, the absence of global measurements for other key BVOCs limits the ability to make comprehensive global BVOC estimations.

Aside from the isoprene retrievals discussed above, modeling is the primary approach used to characterize BVOC emissions across space (Guenther, 2013). The MEGAN model (Guenther *et al*., 2012) has been widely applied to simulate BVOC emissions regionally and globally (Sindelarova *et al*., 2022; DiMaria *et al*., 2023). It improves emission estimates by considering key canopy environmental factors, such as light extinction, leaf area index, leaf age, and the proportion of sun and shade leaves (Niinemets *et al*., 2013). Indeed, these environmental factors are closely linked to photosynthetic activity, which can be detected through photosynthetic optical signals (detailed discussion in The connection between optical remote sensing and BVOC emissions section). Despite progress toward physiological realism, discrepancies between modeled and observed BVOC emissions persist (Guenther *et al*., 2012; Grote *et al*., 2013; Niinemets *et al*., 2013). These discrepancies arise partly due to the reliance on indirect model inputs like generalized emission factors and environmental parameters, which are coarse approximations of natural ecosystems. Importantly, the complex effects of environmental stressors and seasonal dynamics of plant photosynthetic activity on BVOC emissions are difficult to model, and simple parameterizations do not adequately capture this variation (Guenther *et al*., 2012; Grote *et al*., 2013; DiMaria *et al*., 2023; Wang *et al*., 2024). Moreover, gaps in process understanding and limited observations hinder accurate parameterizations, contributing to uncertainty in model predictions and air quality assessment (Arneth *et al*., 2011; Cagliero *et al*., 2021). That is why photosynthetic optical signals such as PRI/CCI and SIF, which can be scaled across landscapes and species to track environmental stresses and photosynthetic dynamics, offer a promising direction for constraining BVOC simulations and improving the accuracy of model predictions.

## The connection between optical remote sensing and BVOC emissions

### Carotenoids and light link foliar reflectance to volatile isoprenoid emissions

Volatile isoprenoids such as isoprene, monoterpenes, and sesquiterpenes share the same biochemical precursors, isopentenyl diphosphate and dimethylallyl diphosphate (DMAPP), with nonvolatile isoprenoids including xanthophyll pigments and other carotenoids (Owen & Peñuelas, 2005; Dudareva *et al*., 2013; Brilli *et al*., 2024). Carotenoids and some volatile isoprenoids, such as isoprene and monoterpenes, also share the same biosynthesis pathway – methylerythritol phosphate (MEP) – which is closely linked with photosynthetic electron transport (Dudareva *et al*., 2013; Dani *et al*., 2014a; Brilli *et al*., 2024). Under stress, potential increases in carotenoid biosynthesis can compete with volatile isoprenoid production for shared substrates – particularly DMAPP – thereby altering emission rates of isoprene or monoterpenes (Owen & Peñuelas, 2005, 2013; Bourtsoukidis *et al*., 2024b). Therefore, changes in carotenoid levels may reflect shifts in substrate allocation between photoprotective pigments and volatile isoprenoids. Consistent with this, correlations between carotenoids and isoprene or monoterpene emissions have been observed (Porcar‐Castell *et al*., 2009; Owen & Peñuelas, 2013; Peñuelas *et al*., 2013). While this metabolic linkage can lead to detectable correlations, the relationship varies depending on factors such as species, developmental stage, and stress conditions.

Foliar visible reflectance, BVOC emissions, and indirectly, SIF, are functionally linked through their shared connection to carotenoids, an important group of isoprenoid metabolites (Fig. 1a). Absorbed solar radiation is partitioned by photosystems into three pathways: (1) electron reducing power fueling photosynthesis and eventual BVOC production (2) dissipation of heat via nonphotochemical quenching (NPQ) to prevent photooxidative damage, and (3) reemission as Chl fluorescence, measured remotely as SIF (Fig. 1a). NPQ increases alongside the reversible xanthophyll cycle – a major group of carotenoid pigments, where violaxanthin is converted to zeaxanthin via antheraxanthin (Fernández‐Marín *et al*., 2021). Changes in foliar reflectance that relate to this occur over a relatively broad spectral range (500–600 nm) in response to changes in illumination, though the precise mechanism for such changes is still not properly understood (Van Wittenberghe *et al*., 2024). Nonetheless, a strong and relatively narrow feature occurs in the vegetation reflectance spectrum centered *c*. 531 nm (Fig. 1b). This feature was quantified in leaf or whole canopy reflectance using PRI (Gamon *et al*., 1992; Peñuelas *et al*., 1995). Many subsequent studies used PRI as a measure of photosynthetic downregulation or LUE, the ratio of GPP to absorbed photosynthetically active radiation, at a range of spatial and temporal scales (see Zhang *et al*., 2016, for a review). PRI is typically calculated by normalizing reflectance at 531 nm to a stable reference band at 570 nm:

(Eqn 1) $$\mathrm{PRI}=\left(R531-R570\right)/\left(R531+R570\right)$$

Excess energy that cannot be used for photosynthesis lowers LUE (Gamon *et al*., 1992; Zhang *et al*., 2016), modulating NPQ and therefore PRI, but is also redirected toward isoprenoid production (Peñuelas *et al*., 2015). Hence, BVOC emissions have been hypothesized to covary with the xanthophyll cycle and the PRI, particularly under stress, when both NPQ and BVOC emissions increase (Owen & Peñuelas, 2005; Loreto & Schnitzler, 2010; Filella *et al*., 2018). Balzarolo *et al*. (2018) found that the total pool of xanthophyll cycle pigments was the primary driver of the PRI–isoprene relationship during the peak of the growing season of *Populus*, coinciding with the highest isoprene emissions. Furthermore, Pollastri *et al*. (2019) provided evidence that isoprene emissions serve as a critical alternative photoprotective mechanism to NPQ under high temperatures, effectively safeguarding plant photosynthesis from thermal stress.

While short‐term (daily) PRI changes mainly reflect xanthophyll pigment dynamics, long‐term variations are predominantly driven by slower changes in total carotenoid content or the Chl‐to‐carotenoid ratio (Gamon & Berry, 2012; Porcar‐Castell *et al*., 2012; Wong & Gamon, 2015; Wong *et al*., 2020). These long‐term changes are closely linked to sustained forms of NPQ, the aforementioned photoprotective mechanism that dissipates excess light energy (Gamon & Berry, 2012; Bag *et al*., 2024). The relationship between carotenoids and NPQ is particularly evident in evergreen species, where an early spring peak of carotenoids and xanthophyll pigments is followed by summer relaxation, reflecting seasonal adjustments to temperature that regulate photosynthesis in such regions (Zhang *et al*., 2019; Rajewicz *et al*., 2023; Bag *et al*., 2024). This photosynthetic seasonality, reflected by carotenoids, can be especially useful in simulating BVOC emissions. A study by Harris *et al*. (2016) demonstrated that total carotenoids, rather than the epoxidation state of xanthophyll pigments in leaves, regulate the negative correlations between PRI and isoprene emissions over longer timescales (days).

The correlation of PRI and BVOC emissions with photosynthetic LUE suggests that PRI could track changes in major plant volatile isoprenoids such as isoprene and monoterpene emissions. Because PRI is closely linked to key physiological processes regulating photosynthetic efficiency at different timescales – short term via the xanthophyll cycle and long term via carotenoids, it has been widely used as a proxy for photosynthetic LUE (Zhang *et al*., 2016). Based on this, Peñuelas *et al*. (2013) showed that PRI was negatively correlated with foliar isoprene from *Populus nigra* and monoterpene emissions from *Quercus ilex* under control and drought conditions, both of which increased as light intensity rose and LUE decreased.

The CCI tracks changes in the total carotenoid pool relative to the Chl pool rather than just the de‐epoxidation state of xanthophyll pigments (DEPS). Those changes better capture photosynthesis over longer seasonal timescales (Wong *et al*., 2020). Wong *et al*. (2020) stated that monitoring such photosynthetic phenology is typically the aim of optical space‐based research, where the usable repeat visit time for a site may be on the order of days or even weeks. Reflecting its heritage in satellite remote sensing (Gamon *et al*., 2016), CCI is defined in terms of NASA Moderate Resolution Imaging Spectroradiometer (MODIS) bands:

(Eqn 2) $$\mathrm{CCI}=\left(\text{Band}\;11-\text{Band}\;1\right)/\left(\text{Band}\;11+\text{Band}\;1\right)$$

where Band 11 is a 10‐nm‐wide band centered at 531 nm (526–536 nm) and is sensitive to xanthophyll cycle pigments; Band 1 is a band centered at 645 nm (620–670 nm), serving as the reference band for CCI.

Filella *et al*. (2018) found that MODIS CCI, which was referred to as ‘PRI (CCI)’ in the study, was significantly correlated with isoprene fluxes in a temperate forest dominated by isoprene‐emitting oak throughout most of the growing season (Table S1). However, this correlation was limited under severe drought, potentially due to increased competition between photorespiration and the MEP pathway for available reducing power and carbon substrates (Dani *et al*., 2014a; de Souza *et al*., 2018).

When integrated into the MEGAN model, the PRI has also improved the accuracy of BVOC estimations over the shorter sub‐seasonal periods (Peñuelas *et al*., 2013), whereas the CCI has improved estimates over longer timescales (Filella *et al*., 2018). Although limited to specific sites, these studies highlight a promising path, which we expound in the Roadmap section, for utilizing remotely sensed carotenoid indices. These indices can now be measured from a growing number of satellite platforms, such as the DLR Earth Sensing Imaging Spectrometer (DESIS; Huemmrich *et al*., 2021), the Global Change Observation Mission‐Climate equipped with the Second‐Generation Global Imager sensor (GCOM‐C/SGLI; Sasagawa *et al*., 2022), and the Hyperspectral Precursor of the Application Mission (PRISMA; Dutta *et al*., 2024).

### Photosynthetic activity links SIF with BVOC emissions

SIF is fluorescence emitted from Chl molecules excited by solar radiation in the range of 650–850 nm (Porcar‐Castell *et al*., 2014). Though Chl fluorescence is emitted as a spectrum, with two emission peaks *c*. 690 and 760 nm (Fig. 1b), SIF primarily refers to remote sensing signals retrieved using infilling techniques applied within discrete, narrow atmospheric features where the small quantity of SIF radiance (0.5–3% of absorbed PAR; Porcar‐Castell *et al*., 2014) is relatively strong due to absorption of solar irradiance within these features (Porcar‐Castell *et al*., 2021). On the ground, Chl fluorescence is also measurable using active radiation techniques such as PAM fluorescence, which plant physiologists have used for decades to characterize processes such as NPQ and the related photochemical quenching of excitation energy (Porcar‐Castell *et al*., 2014; Gu *et al*., 2019; Martini *et al*., 2022; Pierrat *et al*., 2024). The linkage between maximum photosynthetic efficiency, measured from a PAM fluorescence system, and monoterpene emission bursts was also observed in a spring recovery study for a boreal evergreen species, *Pinus sylvestris* (Aalto *et al*., 2015).

SIF and BVOCs are both photosynthetic byproducts, highly responsive to environmental factors such as light, temperature, and particularly stress (Guenther *et al*., 2012; Sun *et al*., 2023). The linkage between SIF and BVOCs lies in their shared connection to photosynthetic activity, stress responses, and energy dissipation, particularly through NPQ, which involves changes in xanthophyll pigments and total carotenoids as described in Carotenoids and light link foliar reflectance to volatile isoprenoid emissions section (Fig. 1a,b). SIF, widely applied to assess plant photosynthesis and stress at multiple scales (Porcar‐Castell *et al*., 2021; Sun *et al*., 2023), has also been explored as a potential constraint on BVOC emissions. Recent satellite studies have shown positive correlations between remotely sensed SIF, MEGAN‐simulated isoprene emissions, and satellite HCHO columns at both ecosystem and regional scales (Table S1; Zheng *et al*., 2017; Trimmel *et al*., 2023; Zhao *et al*., 2024). Zheng *et al*. (2017) found that both SIF and HCHO moderately declined in response to drought stress; the reductions were smaller than the declines in photosynthesis and isoprene emission. This is potentially related to contributions of other VOCs, in addition to isoprene, to HCHO production.

The correlation between SIF and BVOCs may weaken under extreme conditions due to differences in the temperature optima of photosynthesis and isoprene emissions (Zhao *et al*., 2024). Under water and/or heat stress, plants shift energy from fluorescence to heat dissipation through NPQ (Porcar‐Castell *et al*., 2014), while simultaneously increasing BVOC production to mitigate oxidative damage (Pollastri *et al*., 2019, 2023). Lower NPQ has been associated with increased isoprene emissions under high temperatures (Pollastri *et al*., 2014, 2019; Ahrar *et al*., 2015), suggesting NPQ may be an important factor mediating the SIF‐BVOC relationship by regulating energy allocation. Studies on boreal evergreens have shown that SIF correlates with xanthophyll de‐epoxidation state (Magney *et al*., 2019) and carotenoid accumulation (Zhang *et al*., 2019) across seasons. However, the mechanisms underlying the connections between SIF and BVOC emissions remain unexplored, underscoring the need for further research.

SIF has rapidly emerged as a global photosynthesis estimator (Porcar‐Castell *et al*., 2021; Sun *et al*., 2023) and this upward trajectory is expected to continue due to dedicated satellite missions such as the FLuorescence Explorer (FLEX) satellite mission (launch expected in 2026; Gómez‐Giráldez *et al*., 2024). Notably, FLEX is designed for simultaneous carotenoid and xanthophyll retrievals (Van Wittenberghe *et al*., 2024), offering new opportunities to connect PRI/CCI and SIF with BVOC emissions in future studies (Fig. 1c).

In terms of advantages and disadvantages, unlike the carotenoid indices, which have a direct functional link to BVOC emissions through the shared biosynthetic pathway, SIF is indirectly linked to BVOC emissions via photosynthetic activity (i.e. GPP). Although SIF lacks the direct functional linkage to BVOCs like PRI, the connection between SIF and BVOC emissions is likely influenced by shared SIF and BVOC‐sensitive structural factors, such as absorbed photosynthetically active radiation or fractional vegetation cover, rather than by photosynthesis downregulation alone (Porcar‐Castell *et al*., 2021; Sun *et al*., 2023). Currently, satellite‐derived SIF products are more abundant than those for PRI or CCI, with more expected in the near future.

## Current uncertainties and future empirical research directions

### Uncertainties under single or combined abiotic climate‐related stress

**Fig. 2:**
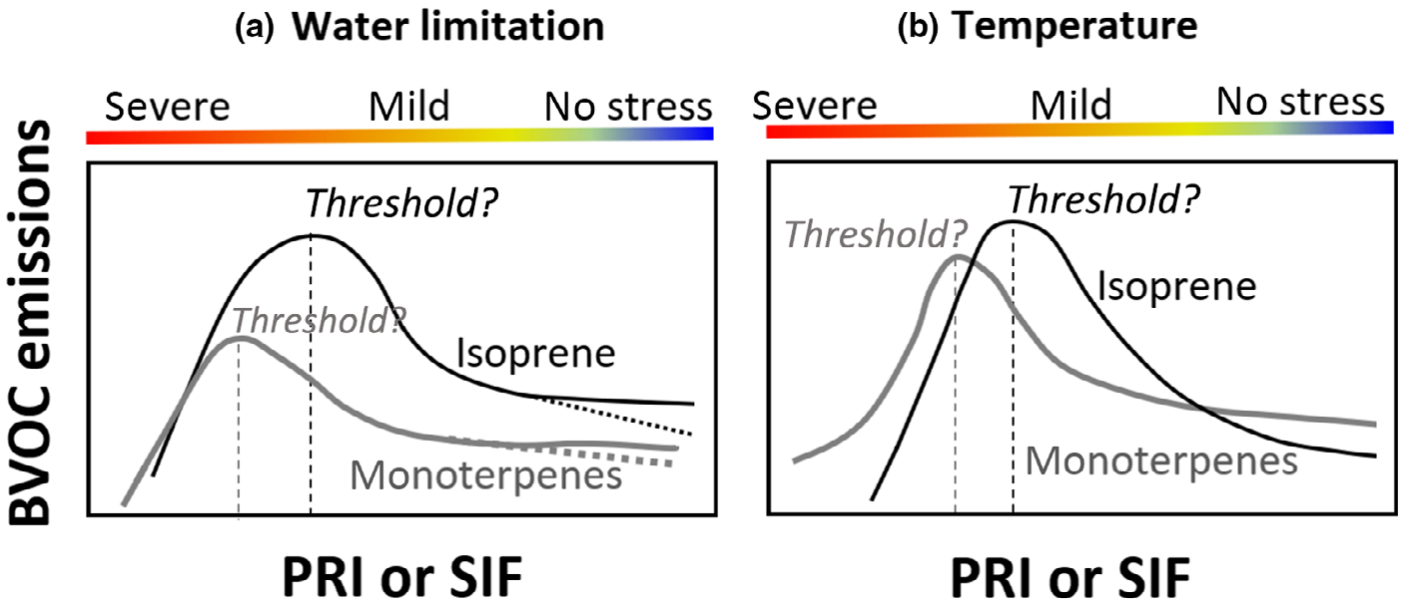
Conceptual models illustrating the relationship between photochemical reflectance index (PRI) or sun‐induced fluorescence (SIF) and biogenic volatile organic compound (BVOC) emissions in response to water (a) and heat (b) stress. The black and gray smooth lines represent correlations with isoprene and monoterpene emissions, respectively. In (a), from no stress to a mild water stress period, isoprene and monoterpene emissions can either remain stable (solid lines) or slightly increase (dotted lines). Question marks represent the uncertainties in specific thresholds that lead to decoupled correlation between PRI or SIF and BVOC emissions.

One of the key knowledge gaps in estimating BVOC emissions is to identify the specific threshold of water or heat stress that triggers significant changes in BVOC emissions (Fig. 2; Box 1). Although environmental stressors are known to influence BVOC emissions, the exact points at which water limitation or high temperature affect these emissions are unclear and likely vary among different BVOC types and plant species (Wang *et al*., 2024; Bourtsoukidis *et al*., 2024b). This variability complicates accurate predictions (Bourtsoukidis *et al*., 2024a) and hinders the use of photosynthetic optical signals to constrain BVOC emissions, particularly under severe stress conditions.

Box 1 **Outstanding questions for future research**

- Abiotic stress and BVOC emissions:
  1. What are the specific thresholds of water or heat stress that uniquely impact BVOC emissions and how do these thresholds vary among different plant species?
  2. Under severe water or heat stress, what are the mechanisms leading to the decoupling of PRI from BVOC emissions?
  3. How do changes in SIF under varying stress conditions correlate with BVOC emissions, and can SIF be used as a robust indicator for estimating these emissions?
  4. How do combined water and heat stress interactively influence BVOC emissions and their correlations with photosynthetic optical signals such as PRI/CCI and SIF, and what are the dominant drivers under such conditions?
  5. How do the species with differing physiological strategies respond to co‐occurring environmental stress in terms of BVOC emissions and photosynthetic optical signals?
- Seasonal and ecosystem‐specific dynamics:
  1. How do photosynthetic optical signals, such as PRI/CCI and SIF, capture the seasonal dynamics of monoterpene emissions in boreal evergreen forests, and what factors dominate these changes?
  2. Can photosynthetic optical signals, such as PRI/CCI and SIF, effectively capture the seasonal dynamics of isoprene emission in Amazonian rainforests, considering their high biodiversity and spatial variability?
  3. How do changes in environmental conditions during critical phenological periods, such as the winter–spring transition in boreal ecosystems or the dry‐to‐wet season transition in the Amazon, affect the relationship between photosynthetic optical signals and BVOC emissions?

The relationship between photosynthetic optical signals and BVOC emissions is nonlinear and complex (Fig. 2; Bourtsoukidis *et al*., 2024b). Specifically, under most stress conditions, photosynthesis declines, while BVOC emissions reach peak levels at a certain threshold of water limitation or high temperature before dropping significantly. Furthermore, different BVOC types such as isoprene and monoterpenes respond differently to stress progression (Feng *et al*., 2019). One key reason is that isoprene is mostly directly emitted through the stomata, whereas monoterpene emissions can also be from a storage pool in addition to stress‐induced biosynthesis and emissions (Niinemets, 2010). Under mild water limitation, both isoprene and monoterpene emissions may remain stable (solid lines in Fig. 2a) or increase slightly (dotted lines in Fig. 2a). By contrast, under mild heat stress, both emissions typically respond immediately to increasing temperature, showing steady and exponential increases (Fig. 2b; e.g. Sharkey & Monson, 2014; Jardine *et al*., 2024; Ndah *et al*., 2024; Wang *et al*., 2024; Bourtsoukidis *et al*., 2024a). After a certain threshold of severe water or heat stress, both emissions decrease, with the decline in monoterpene emissions being more gradual and occurring later than the rapid and sharp decrease in isoprene emissions.

The negative correlation between PRI and BVOC emissions under mild water or heat stress (Fig. 2a,b) occurs because NPQ increases alongside DEPS, leading to a decrease in PRI (Zhang *et al*., 2016, 2017a,b). However, under severe heat or water stress, this relationship may decouple. In such cases, photosynthetic assimilation can decrease considerably, and the PRI eventually reaches a minimum as the xanthophyll cycle pigments becomes fully de‐epoxidized. Despite this, isoprene and monoterpene emissions can still be detectable because alternative carbon sources – such as the reassimilation of CO_2_ under photorespiration conditions or extrachloroplastic carbon sources – can continue contributing to their biosynthesis (de Souza *et al*., 2018). Another factor driving this decoupling could be the excess reducing power, which, when not being used by the Calvin cycle, enhances volatile isoprenoid biosynthesis (Morfopoulos *et al*., 2014; Jardine *et al*., 2024).

Relative to the PRI, the relationship between SIF and BVOC emissions under stress is more uncertain (Box 1). This is because SIF reflects broader processes tied to photosynthetic efficiency, photochemical and nonphotochemical quenching, and vegetation structure under varying stress conditions (Porcar‐Castell *et al*., 2014; Martini *et al*., 2022, 2025; Sun *et al*., 2023). Currently, to our knowledge no studies have investigated the mechanistic linkages between SIF and BVOC emissions in controlled conditions. To achieve this, foliar fluorescence (spectral and PAM) could be integrated with CO_2_, H_2_O, and BVOC emissions in the same sample (Magney *et al*., 2017). This highlights the need for more targeted research into the physiological mechanisms connecting SIF and BVOC emissions.

In contrast to the single stressor discussed above, the effects of combined abiotic stressors, such as water limitation and high temperatures, are less understood but are often more relevant in real‐world conditions (Box 1). These interactions further complicate our understanding of BVOC emissions, limiting the applicability of single‐stressor experiments (Loreto & Schnitzler, 2010). For example, a 2018 heatwave‐drought study found that elevated isoprene emissions could not be explained by high temperature alone; moderate drought also played a crucial role (Ferracci *et al*., 2020). Despite recognition of these interactions (Loreto & Schnitzler, 2010; Niinemets, 2010), BVOC models remain underdeveloped in addressing them (Guenther *et al*., 2012), and the links to optical signals in these conditions are lacking (Box 1). Furthermore, long‐term field studies on combined‐stress effects are scarce (Feng *et al*., 2019; Bao *et al*., 2023). Additionally, limited ecosystem‐scale measurements of isoprene fluxes under severe water stress further hinder model parameterization (Sarkar *et al*., 2020). Although some parameters, such as soil moisture and leaf area index, are incorporated in MEGAN (Guenther *et al*., 2012; Sindelarova *et al*., 2022), temperature is still considered the dominant driver of monoterpene emissions in most studies (Guenther *et al*., 2012; Bourtsoukidis *et al*., 2024a). Therefore, further research is needed to improve our understanding of how combined stressors influence BVOC emissions and their relationship with photosynthetic optical signals.

### Uncertainties in seasonal and ecosystem‐specific dynamics of BVOC emissions in boreal and Amazonian forests

The potential of optical signals to capture the seasonal and ecosystem‐specific dynamics of isoprene and monoterpene emissions is largely unexplored (Box 1). Modeling the seasonality of BVOC is a particularly challenging task because of the complicated interdependencies between environmental conditions, photosynthetic productivity, and the emissions themselves (Grote *et al*., 2013; Guenther, 2013; Niinemets *et al*., 2013). This challenge is enhanced for boreal evergreen species – the major monoterpene emitters (Artaxo *et al*., 2022) – which maintain foliage throughout the year even though their photosynthetic activity exhibits seasonality (i.e. photosynthetic phenology), by responding to changing environmental stimuli. Notably, carotenoid‐related vegetation indices such as CCI and SIF can capture these photosynthetic changes via their dynamic connection to photosynthesis (Pierrat *et al*., 2024). The extent of the connections between boreal zone photosynthetic phenology, optical remote sensing, and BVOC emissions has yet to be elucidated, and is an exciting avenue of future research (see Box 1).

In addition to boreal evergreen ecosystems, the Amazonian rainforest has been the primary focus of most BVOC studies in tropical regions and stands as one of the most globally significant sources of isoprene (Yáñez‐Serrano *et al*., 2020; Artaxo *et al*., 2022; Mu *et al*., 2022). The strong seasonality in Amazonian isoprene emissions – driven by temperature, solar radiation, and leaf phenology – results in high dry‐season emissions due to increased temperature stress and declined emissions in the wet season due to forest biomass loss (Yáñez‐Serrano *et al*., 2020; Alves *et al*., 2023). Notably, SIF has been used to probe this Amazon dry season issue (Doughty *et al*., 2019), but we are not aware of any studies that have focused on connecting the seasonality of SIF and isoprene emissions in this region. Additionally, Amazon's large biodiversity further complicates the quantification of BVOC dynamics at broad spatial and temporal scales (Yáñez‐Serrano *et al*., 2020; Llusià *et al*., 2024). Studying BVOC emissions in the Amazonian rainforest also faces difficulties due to logistical barriers in conducting leaf or tower‐based measurements and limited data on temporal and spatial variability (Yáñez‐Serrano *et al*., 2020).

While these two ecosystems do not encompass the full diversity of global vegetation, they are among the most significant sources of isoprene and monoterpene emissions. They also highlight distinct, unresolved challenges in linking BVOC seasonality to photosynthetic optical signals. Therefore, advancing our understanding within these key ecosystems can serve as a crucial first step before attempting to generalize or scale up remote sensing approaches for estimating BVOC emissions on a global scale. Furthermore, significant uncertainties arise from species‐level variation within these ecosystems. Different plant species vary widely in their dominant BVOC types, emission capacities, and photosynthetic traits. Since PRI and SIF reflect photosynthetic activity and energy partitioning, the strength and nature of their relationship with BVOC emissions depend on how tightly emissions are coupled to photosynthesis. Even among species that emit isoprene – generally considered closely linked to light and photosynthetic processes – this relationship can differ substantially due to differences in emission rates and how emissions are linked to photosynthetic activity. Although direct BVOC flux measurements using techniques like PTR‐MS or GC‐MS can reduce these uncertainties, such measurements are rarely conducted at large scales. Therefore, remote sensing remains a critical tool for inferring BVOC dynamics, emphasizing the need to better understand species‐specific emission traits and their optical signatures to improve model accuracy from local to global scales.

### Observational uncertainty at multiple scales

A rigorous understanding of observational uncertainty is crucial for accurately relating BVOCs to photosynthesis via remote sensing techniques. First, even direct measurements of BVOC emissions are highly susceptible to significant errors due to their volatile nature (Niinemets *et al*., 2010). Second, when considering multiple individuals, representation error must be acknowledged because different chemotypes can exist within the same species (Bäck *et al*., 2011). Third, it is important to note that remote sensing estimates of BVOCs are typically produced through inverse modeling, and satellite PRI/CCI and SIF also have significant uncertainties highlighted below.

When using satellite data, a critical issue arises from the coarse spatial resolution of atmospheric remote sensing observations. For example, Fu *et al*. (2019) developed CrIS isoprene retrievals at nadir ground pixel diameter of 14 km with scanning over a 2200 km swath width. Although BVOCs are less abundant than other atmospheric constituents, the spatial mismatch caused by transport processes must also be considered when relating emissions to surface processes. Chemical transport models can help mitigate this issue and potentially back‐project emissions to their sources.

Satellite SIF and PRI/CCI retrievals do not rely on the full physics‐based inverse approach commonly used in atmospheric chemistry and BVOC retrievals (Fu *et al*., 2019). However, it is essential to properly account for atmospheric effects to obtain accurate surface values in these data (Porcar‐Castell *et al*., 2021). An interesting aspect connecting BVOC remote sensing and SIF is that satellite SIF retrievals have opportunistically utilized atmospheric chemistry sensors due to the high spectral resolution required for SIF (Mohammed *et al*., 2019). This has meant that SIF data has been limited by the coarse ground resolution inherent in many atmospheric sensors, a challenge that ESA's FLEX mission aims to address. A further connection between SIF and atmospheric sounding is that some instruments, such as GOME‐2, used in early SIF studies, can simultaneously estimate formaldehyde – a proxy for isoprene – and carotenoid‐sensitive visible reflectance region. It remains unclear whether this connection has been fully explored.

Beyond atmospheric effects, several other issues must be considered regarding uncertainties in optical remote sensing data. One key point is that artificial satellite data are not direct measurements like field data, but the result of complex retrieval processing chains that probably involve modeling and have multiple sources of uncertainty. Within this text, a key factor for SIF is the magnitude of incident irradiance, since, unlike CCI or PRI, SIF in its basic form is unnormalized for irradiance (Mohammed *et al*., 2019). Furthermore, canopy reflectance and SIF – which can be measured in the field using proximal systems as well as from satellite platforms – are strongly dependent on the relative solar and sensor viewing angles (Pierrat *et al*., 2024). These dependencies are governed by canopy structure and shadowing. A subtle but important mechanism is that the shaded parts of the canopy respond physiologically to the reduced irradiance, which then feeds back into the optical measurement. This phenomenon prompted the development of multiangular PRI methodologies designed to disentangle the effects of structural shadowing from actual photosynthetic downregulation (Hall *et al*., 2008).

It is also important to emphasize that, although incident irradiance is not considered a stressor in the same way as water limitation or heat, the PRI (but not foliar reflectance in general) and SIF both depend on absorbed photosynthetically active radiation physiologically, as discussed above for PRI. At the foliar level, leaves adapt to both their longer term light environments and instantaneous irradiance conditions. This influence has been observed in photosynthetically related leaf optical properties, such as Chl fluorescence emission spectra (Rajewicz *et al*., 2023) and PRI (Gamon & Berry, 2012).

Notably, photoprotective isoprenoid BVOC emissions, including isoprene and monoterpenes, are closely linked to excess light and associated thermal energy dissipation mechanisms. Remote sensing from the top of the canopy is particularly well suited to capture such photoprotective signals, as it primarily samples sunlit upper leaves where light stress is most intense and BVOC emissions are often more active. An interesting hypothesis, therefore, is that spatial variation in foliar optical properties should correlate with spatial variation in photosensitive isoprenoid BVOC emissions, provided other factors – such as chemotypic variability and measurement errors – are properly controlled.

## Roadmap

The functional links between photosynthetic optical signals and BVOC emissions have the potential to improve global emission estimates. However, before this can be realized, targeted experiments and field observations are necessary to address the uncertainties outlined in Current uncertainties and future empirical research directions. Additionally, a modeling strategy is required to upscale these insights effectively. We conclude this Viewpoint by discussing both aspects.

### Experimental and observational requirements

**Fig. 3:**
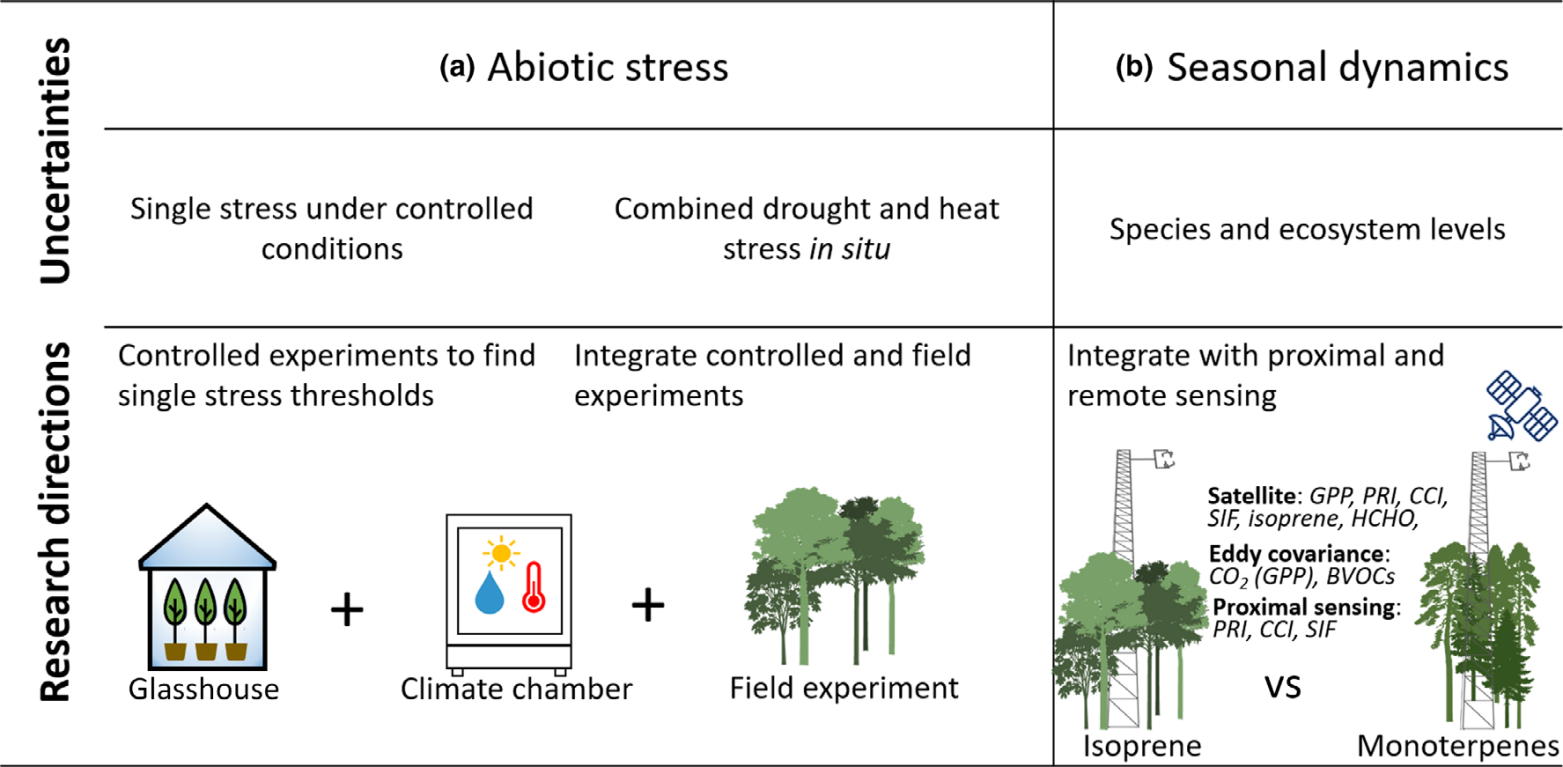
Summary of research uncertainties and future directions for using photosynthetic optical signals to constrain biogenic volatile organic compound (BVOC) emissions (a) under abiotic stress and (b) across seasonal dynamics. CCI, Chl/carotenoid index; GPP, gross primary productivity; HCHO, formaldehyde; PRI, photochemical reflectance index; SIF, sun‐induced fluorescence.

Controlled experiments are essential to understand how abiotic stressors influence BVOC emissions and their relationship with optical signals (Fig. 3a). These experiments would manipulate temperature and water conditions in climate chambers and glasshouses to simulate single or combined stresses. Measurements of BVOC emissions and optical signals, such as PRI and SIF, could then be taken during these trials (Fig. 3a). Nevertheless, validating these findings in natural conditions is crucial. Hence, ecosystem‐scale drought experiments (Jiang *et al*., 2020; D'Odorico *et al*., 2021; Rissanen *et al*., 2022) are also needed to provide valuable insights at scales larger than an individual.

For analyzing seasonal dynamics, it is essential to compare photosynthetic optical signals with satellite‐retrieved BVOC data (e.g. formaldehyde or CrIS‐based isoprene) at key phenological stages, such as the winter–spring transition in boreal regions (Gamon *et al*., 2016) or the dry‐to‐wet transition in the Amazon (Doughty *et al*., 2019; Alves *et al*., 2023). Given that the environmental responses of isoprene and monoterpene emissions are complex and still under debate (Loreto & Schnitzler, 2010; Feng *et al*., 2019), conducting comprehensive *in situ* measurements on at least two contrasting ecosystems each dominated by either isoprene or monoterpenes (Fig. 3b) would significantly enhance understanding. This could involve continuous leaf‐level BVOC and gas exchange measurements using species‐specific enclosures, complemented by proximal sensing of optical properties (VIS–NIR reflectance and SIF). These data streams could then be integrated with ecosystem‐scale measurements like eddy covariance to explore seasonal relationships and refine models (Fig. 1c; Aalto *et al*., 2015; Nichol *et al*., 2019; Artaxo *et al*., 2022).

### Scaling and modeling considerations and broader applications

Biogenic emission models such as MEGAN offer the potential to leverage optical remote sensing data for improving emission estimates. For instance, Filella *et al*. (2018) combined MEGAN's isoprene predictions with an empirical PRI/CCI‐based approach, outperforming the standard model. An ideal next step is to directly integrate remote sensing data within such models by adjusting the emission activity factor, $\gamma_{i}$:

(Eqn 3) $$F_{i}=\gamma_{i}\sum \varepsilon_{i,j}\chi_{j}$$

Here, $F_{i}$ is the emission for chemical species i, $\varepsilon_{i,j}$ is an emission factor under standard conditions for chemical species i and vegetational type *j*, and *χ*_*j*_ is a factor accounting for the area coverage of a vegetation type within a grid cell.

The emission activity factor ($\gamma_{i}$) could potentially be scaled using remote sensing‐derived variables like photosynthetic LUE, which are related to PRI/CCI. For example, Zhang *et al*. (2022) demonstrated how microwave remote sensing data could refine MEGAN's predictions by incorporating soil moisture information in a similar way. An even more promising approach involves fusing multiple remote sensing data streams – such as optical measurements (SIF), microwave data, and thermal infrared – each improving specific aspects of emission activity parameterization (e.g. water stress detection, phenology, leaf area).

Beyond emission estimates, optical remote sensing also holds promise for better quantifying net ecosystem exchange (NEE) – the difference between ecosystem carbon uptake and release. NEE, and the derived parameters GPP and ecosystem respiration, are calculated from CO_2_ fluxes measured via eddy covariance, but a small portion of this flux results from BVOC emissions. Under stress conditions, BVOC emissions can increase significantly while photosynthesis declines, amplifying their contribution to the carbon budget. For example, Sharkey & Loreto (1993) reported that isoprene emission from kudzu plants could reach about two‐thirds of the photosynthetic carbon fixation under certain stress scenarios. Additionally, processes like photorespiration – competing for photosynthetic reducing power – are difficult to measure directly but can influence BVOC emissions, especially under stress (Peñuelas & Llusià, 2002; Jardine *et al*., 2014; Dani *et al*., 2014b). Correlating optical signals with BVOC fluxes could help close these gaps in carbon budget understanding.

In summary, there are clear opportunities to link optical remote sensing with BVOC emissions, leveraging current measurement technologies and models. However, a major source of uncertainty remains regarding the broader applicability and generalization of these approaches, given the variability in chemotypes across species, ecosystems, and regions. Addressing this challenge requires targeted empirical research. Furthermore, integrating *in situ* measurements with satellite data – such as co‐registered BVOC and optical proxies like SIF and PRI – could significantly enhance our ability to monitor emissions at large scales and deepen our understanding of their interactions with photosynthesis, atmospheric chemistry, and climate dynamics.

## Competing interests

None declared.

## Author contributions

CZ formulated the main ideas and concepts of the manuscript and led the writing of the original draft. JA contributed to the writing and revision of the manuscript. JB, JP, DL, IF and AP‐C discussed the design and ideas and contributed significantly to the revision of the manuscript.

## Disclaimer

The New Phytologist Foundation remains neutral with regard to jurisdictional claims in maps and in any institutional affiliations.

## Supporting information


**Table S1** Summary of studies that used PRI and SIF to estimate BVOC emissions.
Please note: Wiley is not responsible for the content or functionality of any Supporting Information supplied by the authors. Any queries (other than missing material) should be directed to the *New Phytologist* Central Office.
