# Supplementary material for: Connecting optical remote sensing of plant photosynthesis with biogenic volatile organic compound emissions
Source: New Phytol. 2025 Aug 31;248(2):494–506. doi: 10.1111/nph.70504 (PMC12445812; doi:10.1111/nph.70504)
Supplement: Supplementary file 1 — Table S1 Summary of studies that used PRI and SIF to estimate BVOC emissions. Please note: Wiley is not responsible for the content or functionality of any Supporting Information supplied by the authors. Any queries (other than missing material) should be directed to the New Phytologist Central Office. [file NPH-248-494-s001.pdf]

***New Phytologist* Supporting Information**

Article title: Connecting optical remote sensing of plant photosynthesis to biogenic volatile organic compound emissions

Authors: Chao Zhang, Jaana Bäck, Josep Peñuelas, Daijun Liu, Iolanda Filella, Albert Porcar-Castell, Jon Atherton

Article acceptance date: 28 July 2025

The following Supporting Information is available for this article:

Table S1 Summary of studies that used PRI or SIF to estimate BVOC emissions.

References

Table S1 Summary of studies that used PRI or SIF to estimate BVOC emissions

Optical indices	Literature	BVOC types	Plant species	Spatial scale	Growth conditions	Environmental context
PRI	Peñuelas *et al*. (2013)	Isoprene emission rates	*Populus nigra*	Leaf	4-years old potted seedlings	Experimental drought
		Monoterpene emission rates	*Quercus ilex*	Leaf	4-years old potted seedlings	Experimental drought
	Harris *et al*. (2016)	Isoprene emission rates	*Salix viminalis*	Leaf	1 to 2 years old potted seedlings	Three natural light conditions: sun, shade, half shade
	Filella *et al*. (2018)	Isoprene emission rates	A temperate deciduous forest, dominated by oaks	Ecosystem	Natural oak	Natural drought
	Balzarolo *et al*. (2018)	Isoprene emission rates	Populus	Leaf, canopy	Managed plantation	Natural light variations within the canopy
SIF	Zheng *et al.* (2017)	HCHO column	A temperate deciduous forest, dominated by oaks	Ecosystem	Natural oak	Natural drought
		Isoprene emission fluxes				
	Trimmel *et al.* (2023)	HCHO mixing ratios	Mixed species in Vienna city and around area	Regional	Vienna city and around area	Natural drought
	Zhao *et al.* (2024)	HCHO column	Mixed species in Northern high latitude	Continental	Natural	Natural
		Isoprene emission fluxes		Ecosystem	Natural	Natural

References:

Balzarolo M, Peñuelas J, Filella I, Portillo-Estrada M, Ceulemans R. **2018**. Assessing ecosystem isoprene emissions by hyperspectral remote sensing. *Remote Sensing* **10**(7): 1–11.

Filella I, Zhang C, Seco R, Potosnak M, Guenther A, Karl T, Gamon J, Pallardy S, Gu L, Kim S, *et al.* 2018. A MODIS photochemical reflectance index (PRI) as an estimator of isoprene emissions in a temperate deciduous forest. *Remote Sensing* **10**: 1–16.

Harris A, Owen SM, Sleep D, Pereira M da G dos S. **2016**. Constitutive changes in pigment concentrations: Implications for estimating isoprene emissions using the photochemical reflectance index. *Physiologia Plantarum* **156**: 190–200.

Peñuelas J, Marino G, Llusia J, Morfopoulos C, Farré-Armengol G, Filella I. **2013**. Photochemical reflectance index as an indirect estimator of foliar isoprenoid emissions at the ecosystem level. *Nature Communications* **4**: 2604.

Trimmel H, Hamer P, Mayer M, Schreier SF, Weihs P, Eitzinger J, Sandén H, Fitzky AC, Richter A, Calvet JC, *et al.* 2023. The influence of vegetation drought stress on formaldehyde and ozone distributions over a central European city. *Atmospheric Environment* **304**: 119768.

Zhao T, Mao J, Simpson WR, De Smedt I, Zhu L, Hanisco TF, Wolfe GM, St. Clair JM, González Abad G, Nowlan CR, *et al.* 2022. Source and variability of formaldehyde (HCHO) at northern high latitudes: An integrated satellite, aircraft, and model study. *Atmospheric Chemistry and Physics* **22**: 7163–7178.

Zheng Y, Unger N, Tadić JM, Seco R, Guenther AB, Barkley MP, Potosnak MJ, Murray LT, Michalak AM, Qiu X, *et al.* 2017. Drought impacts on photosynthesis, isoprene emission and atmospheric formaldehyde in a mid-latitude forest. *Atmospheric Environment* **167**: 190–201.
